# Heterogeneous Differentiation of Human Mesenchymal Stem Cells in 3D Extracellular Matrix Composites

**DOI:** 10.1089/biores.2015.0044

**Published:** 2016-01-01

**Authors:** Jangwook P. Jung, Meredith K. Bache-Wiig, Paolo P. Provenzano, Brenda M. Ogle

**Affiliations:** ^1^ Department of Biomedical Engineering, University of Minnesota–Twin Cities, Minneapolis, Minnesota.; ^2^ Stem Cell Institute, University of Minnesota–Twin Cities, Minneapolis, Minnesota.; ^3^ Masonic Cancer Center, University of Minnesota–Twin Cities, Minneapolis, Minnesota.; ^4^ Institute for Engineering in Medicine, University of Minnesota–Twin Cities, Minneapolis, Minnesota.; ^5^ Lillehei Heart Institute, University of Minnesota–Twin Cities, Minneapolis, Minnesota.

**Keywords:** biomaterials, extracellular matrix, stem cells

## Abstract

Extracellular matrix (ECM) proteins are structural elements of tissue and also potent signaling molecules. Previously, our laboratory showed that ECM of 2D coatings can trigger differentiation of bone marrow-derived mesenchymal stem cells (MSCs) into mesodermal lineages in an ECM-specific manner over 14 days, in some cases comparable to chemical induction. To test whether a similar effect was possible in a 3D, tissue-like environment, we designed a synthetic-natural biomaterial *composite.* The composite can present whole-molecule ECM proteins to cells, even those that do not spontaneously form hydrogels *ex vivo*, in 3D. To this end, we entrapped collagen type I, laminin-111, or fibronectin in ECM composites with MSCs and directly compared markers of mesodermal differentiation including cardiomyogenic (*ACTC1*), osteogenic (*SPP1*), adipogenic (*PPARG*), and chondrogenic (*SOX9*) in 2D versus 3D. We found the 3D condition largely mimicked the 2D condition such that the addition of type I collagen was the most potent inducer of differentiation to all lineages tested. One notable difference between 2D and 3D was pronounced adipogenic differentiation in 3D especially in the presence of exogenous collagen type I. In particular, *PPARG* gene expression was significantly increased ∼16-fold relative to chemical induction, in 3D and not in 2D. Unexpectedly, 3D engagement of ECM proteins also altered immunomodulatory function of MSCs in that expression of *IL-6* gene was elevated relative to basal levels in 2D. In fact, levels of *IL-6* gene expression in 3D composites containing exogenously supplied collagen type I or fibronectin were statistically similar to levels attained in 2D with tumor necrosis factor-α (TNF-α) stimulation and these levels were sustained over a 2-week period. Thus, this novel biomaterial platform allowed us to compare the biochemical impact of whole-molecule ECM proteins in 2D versus 3D indicating enhanced adipogenic differentiation and *IL-6* expression of MSC in the 3D context. Exploiting the biochemical impact of ECM proteins on MSC differentiation and immunomodulation could augment the therapeutic utility of MSCs.

## Introduction

Utilization of mesenchymal stem cells (MSCs) in the clinic and attempts to drive mesodermal differentiation of MSCs *in vitro* would benefit from enhanced understanding of the impact of 3D, exogenous extracellular matrix (ECM) on MSC state. Indeed MSCs are a heterogenous lot, primed to mature to several distinct mesodermal states or to remain multipotent depending on the local environment (e.g., native tissue, recipient tissue or tissue culture scenario). The local environment contains soluble signaling factors, other cells or cell types, and ECM, and the bulk of past literature reports pertain to soluble factor stimulation with respect to MSC state. More recently, an appreciation of the role of ECM in MSC specification has emerged including work of our laboratory in 2009 showing the presentation of ECM proteins on 2D culture plastic to MSCs is capable of triggering differentiation into multiple lineages at levels comparable to soluble factor induction.^1^ This result is perhaps not now surprising as the multifaceted functions of ECM continue to emerge.^2–4^

ECM proteins, aside from providing support for cells and tissues, are important modulators of cell signaling during development, in the stem cell niche, and in the tumor microenvironment. ECM proteins are large and complex, and contain highly conserved regions.^5^ ECM proteins act as effective ligands to initiate cell signaling usually via integrin receptors. They may also act as a depot to controllably bind or release growth factors that drive a multitude of cell signaling events. Despite the multifunctionality of ECM proteins in guiding cell behavior such as proliferation and migration, systematic understanding of the role of ECM in stem cell differentiation is somewhat limited in conventional pseudo-3D culture platforms.^6^ Covalent conjugation^7^ or adsorption^8^ of ECM proteins on 2D surfaces have been simple and convenient platforms to create microenvironments for stem cell differentiation. Efforts to create more physiologically relevant 3D microenvironments^9^ were carried out by controlling matrix mechanics,^10^ porosity,^11^ or cell aggregates^12^ and by forming scaffolds with collagen, Matrigel^®^, decellularized ECM, and ECM-mimicking oligopeptides.^13^ Although the distinction between stem cell differentiation in 2D versus 3D was not ignored,^14–16^ a direct comparison of ECM presentation in 2D versus 3D has been rarely evaluated.^17^ Here, we utilized a recently developed 3D biomaterial platform^18^ to compare MSC differentiation to cardiomyogenic, osteogenic, adipogenic, and chondrogenic lineages in 2D versus 3D.

**FIG. 1.**
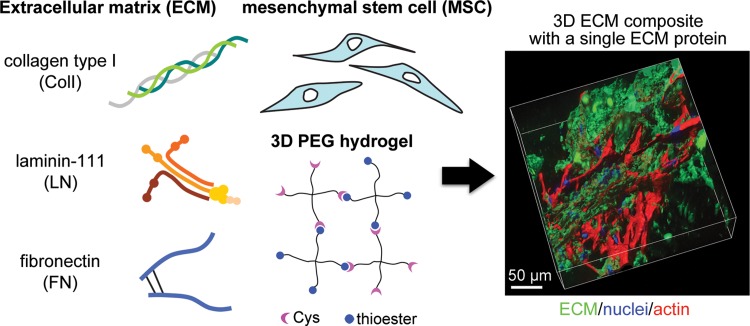
Schematic for production of extracellular matrix (ECM) composites with either collagen type I, laminin-111, or fibronectin and mesecnhymal stem cell (MSC) in four-armed poly(ethylene glycol) (PEG) hydrogels. ECM protein, MSC, and PEG-precursor are the core components of a 3D ECM composite in the article. PEG-precursors are functionalized with either cysteine (Cys) or thioester, forming native amide bond (CONH) (i.e., native chemical ligation). A representative z stack obtained from a collagen type I composite after 21 days of human MSC culture (right).

We created “composites” by incorporating ECM protein(s) and stem cells in poly(ethylene glycol) (PEG) hydrogels, which are cross-linked via formation of native amide bonds.^19^ This chemoselective cross-linking allows the presentation of ECM proteins without any modification or conjugation to PEG chains and maintains consistent overall stiffness and diffusion in the presence of exogenous ECM proteins. This platform enables biochemical engagement of ECM proteins to MSCs in 3D, largely excluding other biophysical stimuli. Here, human embryonic stem cell (hESC)-derived MSCs (hMSCs)^20,21^ were encapsulated in ECM composites with 2 mg/mL of either collagen type I, laminin-111, or fibronectin (Fig. 1). MSC functionality was assessed via gene and protein expression of lineage markers associated with cardiomyogenesis, adipogenesis, osteogenesis, and chondrogenesis and of cytokines associated with immunomodulation.

## Materials and Methods

Detailed methods are provided in the Supplementary Materials and Methods section.

### Culture of human mesenchymal stem cells and formation of ECM composites and ECM films

Mesenchymal stem cells were derived from H1 (hESC, a gift from Dr. Peiman Hematti, University of Wisconsin-Madison)^20^ and markers indicative of the MSC phenotypes (CD73, CD90, and CD105) were probed by flow cytometry (Supplementary Fig. S1). Human MSC (hMSC) were encapsulated in ECM composites^18^ or cultured on 2D ECM films^1^ using protocols previously published (Supplementary Materials and Methods).

### Imaging ECM composites using multi-photon laser scanning microscopy

At days 1, 14, and 28, ECM composites were fixed and stained with previously published protocols (Supplementary Materials and Methods).^18^ ECM proteins, nucluei, and actin filaments were visualized with a 40× (NA = 0.8) objective lens by using a mode-locked Ti:Sapphire laser on a multi-photon laser scanning microscope (MPLSM; Bruker Nano). 3D reconstructions were generated using Imaris 7.5.2 software (Bitplane, Inc.). To further confirm the expression of FABP4 and PPAR-γ proteins from H1 MSCs after 28 days, ColI composites were incubated overnight at 4°C with rabbit anti-FABP4 (cat# PA5-30591; Thermo Scientific, 1:100 dilution in the blocking solution) or rabbit-PPARγ (cat# PA3-821A; Thermo Scientific, 1:100 dilution in blocking solution) antibodies, which were probed by goat anti-rabbit FITC (cat# 31583; Pierce, 1:200 dilution in blocking solution) for 45 min.

### Quantitative real-time polymerase chain reactions

RNAs were extracted from hMSC culture or ECM composites using a standard TRIzol^®^ method (Supplementary Materials and Methods). Complementary DNA (cDNA) was synthesized following the instructions from the Maxima First Strand cDNA Synthesis Kit (cat# K1642; Thermo Scientific). Each lineage primer sequence is provided in Table 1. Immunomodulatory gene primers (*IL-6* and *IDO*) were purchased from Biorad (cat# 10025636). Primer efficiencies were extracted from RealPlex^2^ software and verified with melting curves. Each lineage standard curve was calculated using terminally differentiated cells and corresponding primers. The standard curve method was employed to determine copy numbers of each lineage and *GAPDH* gene. The lineage gene expression level was determined as the copy number of lineage gene of interest normalized to that of *GAPDH* at each time point.

**Table 1.** **qRT-PCR Primer Sequences for hMSC Lineage Markers**

Target gene	Primer sequence (5′ → 3′)	Amplicon size (bp)
*SPP1*	FOR: TTCGCAGACCTGACATCCAGTACC	70
	REV: TCCTCGCTTTCCATGTGTGAGG	
*PPARG*	FOR: AGCCTCATGAAGAGCCTTCCAAC	122
	REV: TCTCCGGAAGAAACCCTTGCATC	
*ACTC1*	FOR: GCTTCCGCTGTCCTGAGA	61
	REV: ATGCCAGCAGATTCCATACC	
*SOX9*	FOR: TTCCGCGACGTGGACAT	77
	REV: TCAAACTCGTTGACATCGAAGGT	
*GAPDH*	FOR: TTAAAAGCAGCCCTGGTGAC	144
	REV: CTCTGCTCCTCCTGTTCGAC	

hMSC, human mesenchymal stem cells; qRT-PCR, quantitative real-time polymerase chain reaction.

### hMSC chemical induction

Standard chemical reagents were used for cardiomyogenic,^22^ osteogenic,^21^ adipogenic,^21^ and chondrogenic^23^ differentiation (Supplementary Materials and Methods).

### Stimulation of hMSCs with IFN-γ and TNF-α

hMSCs suspended in α-MEM medium were plated to six-well plates at a density of 1.4 × 10^4^ cells/mL and were kept at 37°C/5% CO_2_ for 20 h. Stimulation media were prepared with 100 ng/mL of IFN-γ (cat# 300-02; Peprotech) or 50 ng/mL of TNF-α (cat# 68-8786-82; eBioscience) in α-MEM medium. After 20 h of culture, the medium in the six-well plates was replaced with stimulation media, which was added to the experimental wells and α-MEM medium in the control wells. The plates were kept at 37°C/5% CO_2_ and hMSCs were collected at 4 and 24 h.

### Statistical analyses

For comparison of expression levels of various differentiation or immunomodulatory markers of hMSCs from ECM composites, one-way ANOVA with the Tukey's HSD *post hoc* test, Fisher's LSD *post hoc* test, or Student's *t*-test (α = 0.05) was performed. All tests were performed using Statistical Analysis System (SAS Institute, Inc.).

## Results

### hMSCs in ECM composites exhibit morphologies influenced by ECM engagement in 3D

**FIG. 2.**
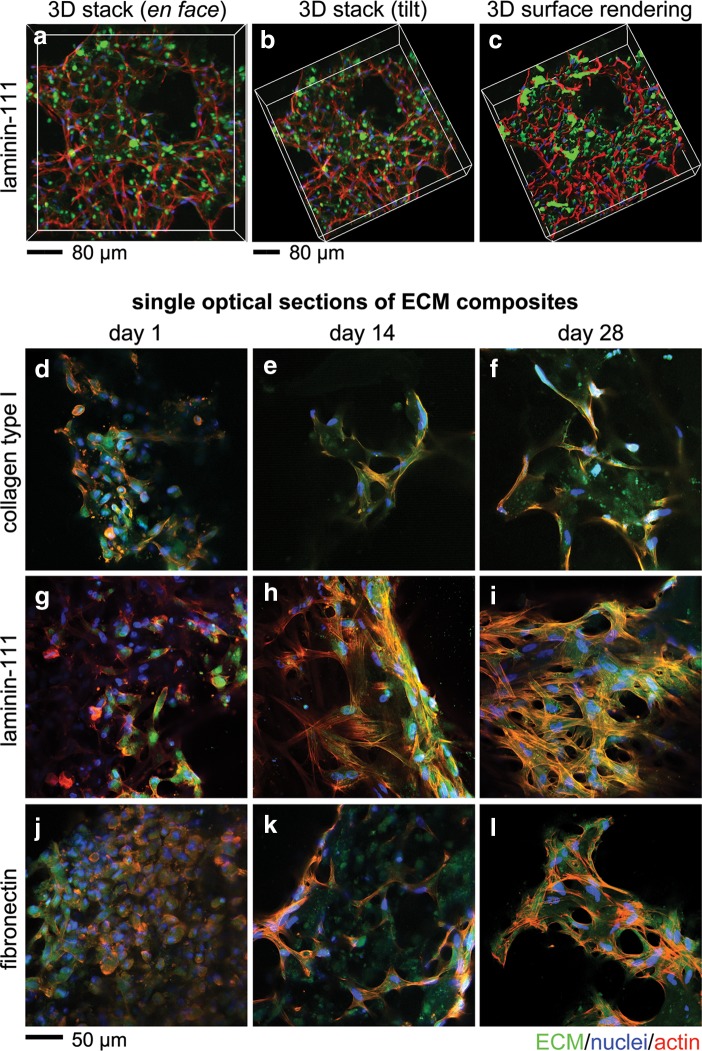
Morphology and engagement of human MSCs (hMSCs) with ECM of 3D composites over time. Multi-photon laser scanning microscopy (MPLSM) was used to optically section (at 1 μm interval) microenvironments of LN composites containing hMSCs to convey the three-dimensionality of the microenvironments within the composite **(a–c,** 161 planes). Z stacks of images were reconstructed from raw images **(a, b)** to solid objects using Imaris software to best view cell–ECM interactions **(c)**; scale bar 80 μm. Single optical sections of ECM composites at days 1, 14, and 28 after composite formation. ColI (collagen type I) composites **(d–f)**, LN (laminin-111) composites **(g–i)**, and FN (fibronectin) composites **(j–l)**. ECM proteins (green), nuclei (blue), and actin filaments (red); Scale bar 50 μm.

ECM proteins, provided exogenously at the time of composite formation, distinctively impacted the distribution and morphology of hMSCs in 3D. After 3 weeks in culture, hMSCs in laminin (LN) composites exhibited reticulate structures and were found to adhere in multiple dimensions to the network architecture of LN (Fig. 2a–c). The 3D surface rendering of z stacks at over 162 μm showed that hMSCs were engaged with LN throughout the PEG-entrapped microenvironments of composites (Fig. 2b, c). Similar 3D engagement with ColI was observed at this time point, but with cells assuming an elongated morphology and cell-ECM structure, usually globular in nature as previously reported from our laboratory.^18^ We further probed the engagement of hMSCs with surrounding ECM at 1, 14, and 28 days of culture utilizing MPLSM. Since each field of view was not taken from a flat, 2D region of interest, some optical sections show narrower aggregate structures (e.g., Fig. 2e, f or l) than others (e.g., Fig. 2i or j). All composites showed globular microenvironments at day 1 with high hMSC to ECM ratios (Fig. 2d, g, j). At later time points, composites evolved to contain a slightly lower hMSC to ECM ratio likely due to accumulating endogenous ECM and associated decrease in hMSC proliferation.^18^ By day 28, distinct hMSC morphologies and corresponding microenvironments emerge. ColI composites showed globular aggregation with significant hMSC spreading (Fig. 2f). LN, as noted above, assumes a reticulate or mesh-like hMSC-ECM distribution (Fig. 2i). A higher number of hMSCs attached to fibronectin (FN) than other ECM, perhaps attributed to increased cell-binding domains^24^ relative to ColI or LN. FN composites showed fewer numbers of cells per microenvironment and smaller total volume per microenvironment (Fig. 2l) than LN composites. Collectively, ECM proteins exogenously supplied in 3D composites were able to modulate hMSC-ECM engagement. Indeed, cell–ECM interactions appeared quite similar between groups at day 1, but quickly changed over time in distinctive ways according to ECM type.

### Differentiation of MSCs, especially adipogenesis, is initiated in 3D ECM composites

**FIG. 3.**
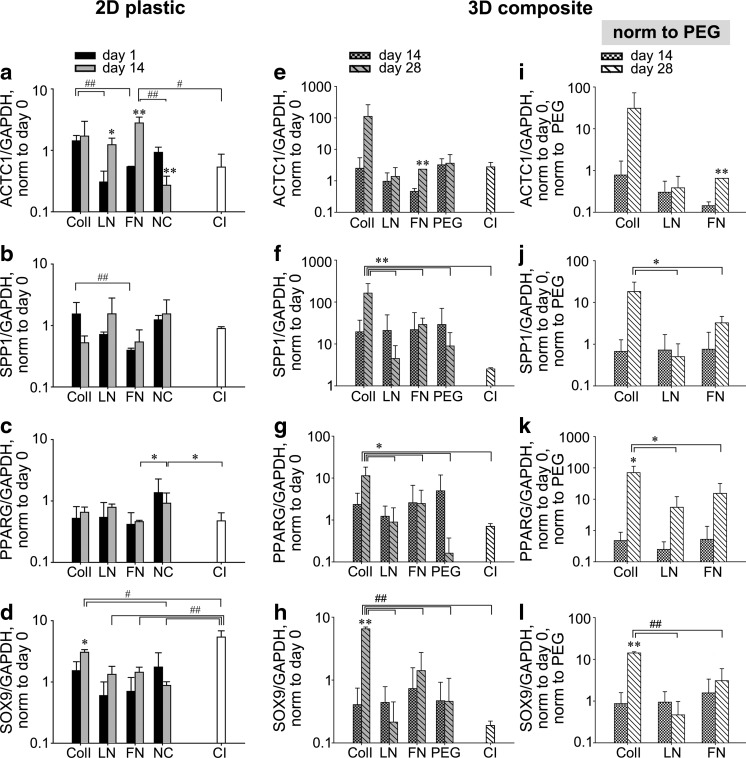
Multi-lineage differentiation of hMSCs on 2D ECM films and in 3D ECM composites. The expression of *ACTC1* (cardiomyogenic), *SPP1* (osteogenic), *PPARG* (adipogenic), and *SOX9* (chondrogenic) on 2D ECM films was measured using quantitative real-time polymerase chain reaction (qRT-PCR). Chemical induction (CI) without 2D ECM films, performed in parallel with cultures on 2D ECM films **(a–d)**. The expression of *ACTC1*, *SPP1*, *PPARG*, and *SOX9* in 3D composites was probed by qRT-PCR **(e–h)**. The relative expression of each lineage marker was further normalized to the relative expression of composites containing hMSCs and PEG only at each corresponding time point **(i–l)**. CI in 3D composite was performed in tissue culture flasks **(e–h)**. mean ± SD, *n* = 3. ANOVA Tukey's HSD *post hoc* test, ##*p* < 0.01 and #*p* < 0.05. ANOVA Fisher's LSD *post hoc* or Student's *t*-test, **p < 0.01 and *p < 0.05. * or ** without brackets between the time points in the group.

Previously, our laboratory reported that 2D ECM coatings alone were able to initiate differentiation of bone marrow-derived MSCs into cardiomyogenic, osteogenic, and adipogenic lineages at levels comparable to chemical induction.^1^ Unclear at that time was whether or not differentiation in the context of ECM was an artifact of the unnatural spreading and associated strains of the 2D tissue culture scenario or whether the same could occur in a 3D, tissue-like context. Appropriate technology to directly test this question took several years to develop since many ECM types extracted from the body do not form 3D hydrogels; but we have recently validated such a system.^18^ We use it here to determine whether ECM composites, each containing a single ECM at a fixed concentration of 2 mg/mL and with maintained stiffness^18^ and diffusivity (Supplementary Table S1) could modulate the functional capacity of hMSCs both in terms of differentiation and immunomodulatory potential. For the purpose of comparison, we repeated the 2D studies^1^ here using hMSCs and reexamined the differentiation profile (Fig. 3a–d). In 2D, hMSCs cultured on ECM films of LN and FN showed increased expression of *ACTC1* (i.e., cardiomyogenic differentiation) at day 14 relative to day 1, but only FN stimulated gene expression levels significantly exceeding those of the no construct (NC) control *and* chemical induction (Fig. 3a). Osteogenic differentiation (as discerned by *SPP1* expression) of hMSCs on 2D ECM films generally increased from day 1 to 14 (with the exception of ColI; Fig. 3b) to levels comparable to chemical induction at day 14. None of the 2D ECM films promoted *PPARG* expression associated with adipogenic differentiation. Surprisingly, only the NC control yielded *PPARG* expression slightly, but significantly higher than chemical induction at day 14 (Fig. 3c). Similarly, none of the 2D ECM films promoted *SOX9* expression associated with chondrogenic differentiation to levels approaching that of chemical induction. It should be noted that the chemical induction protocol for chondrogenesis requires a cell pellet that essentially creates a 3D environment, and so perhaps is not the most robust 2D control.

In 3D ECM composites, we found ColI significantly promoted the expression of *SPP1*, *PPARG*, and *SOX9* genes of hMSCs at 28 days relative to 2D chemical induction (Fig. 3e–h). The average expression of *ACTC1* in ColI composite was relatively high while its variation was also large and thus not significantly different from 2D chemical induction at day 28 (Fig. 3e). Osteogenic and adipogenic differentiation in the ColI composite were significantly higher than chemical induction and other ECM composites at day 28 (Fig. 3f, g). In addition, chondrogenic differentiation in ColI composite was significantly increased from day 14 to 28, and was also significantly higher than chemical induction and other ECM composites at day 28 (Fig. 3h). To distinguish the contribution of the 3D environment only (PEG) from the 3D environment plus exogenous ECM on hMSC differentiation, we normalized each expression level of lineage genes to that of the PEG control at each time point (Fig. 3i–l). ColI composites significantly upregulated expression of each lineage gene at day 28 (Fig. 3j–l), except *ACTC1* (cardiomyogenic) (Fig. 3i). Adipogenic and chondrogenic gene expression of hMSCs in ColI composites was also significantly increased from day 14 to 28, respectively (Fig. 3k, l). Taken together, differentiation of ESC-derived MSCs in 3D ColI composites was significantly more effective than 2D chemical induction, while chemical induction of hMSCs was more efficient than 2D ECM films only in chondrogenic induction by 3D cell mass.

**FIG. 4.**
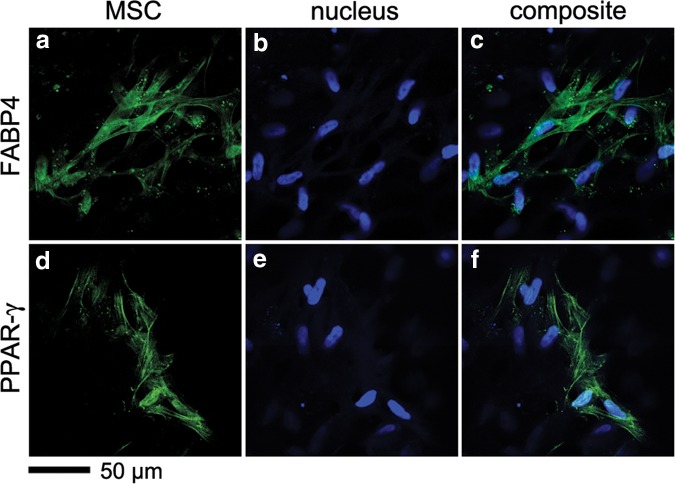
Expression of FABP4 and PPAR-γ proteins in 3D ColI composites after 28 days of culture. Immunofluorescence was visualized by MPLSM. FABP4 **(a–c)** and PPAR-γ **(d–f)**. Adipogenic protein markers (green) and nuclei (blue); scale bar 50 μm.

The most outstanding difference in hMSC differentiation progress between 2D and 3D exogenous ECM engagement was adipogenic differentiation. Minimal *PPARG* expression was detected in 2D ECM films (Fig. 3c), while 3D ECM composites all upregulated *PPARG* gene expression at both 14 and 28 days, especially in composites with ColI (Fig. 3g). The significant upregulation of *PPARG* gene led to query of the expression of adipogenic proteins, FABP4 and PPAR-γ in 3D ColI composites. After 28 days of hMSC culture in ColI composite, hMSCs were stained with antibodies against FABP4 and PPAR-γ using MPLSM (Fig. 4). Over 28 days, hMSCs in ColI composites showed clusters of hMSCs positive for both FABP4 (Fig. 4c) and PPAR-γ (Fig. 4f) protein expression. Staining of both adipocyte-associated proteins was more intense in 3D composites containing collagen type I than that observed in all 2D cultures with ECM films and even with chemical induction where only limited, faint staining was observed. Due to the limited protein staining in the 2D condition, especially with chemical induction, we also exposed an alternative ESC-derived MSC line (H9 MSCs) to chemical induction for adipogenesis (Supplementary Fig. S2a, e).^21^ We found a moderate level of staining for PPAR-γ and FABP4, definitive Oil Red O staining of fat droplets (Supplementary Fig. S2i) and significant upregulation of *PPARG* gene (Supplementary Fig. S2j) suggesting the chemical induction protocol was effective, and that the H1 MSC line is less prone to adipogenesis than other MSC populations such as H9 MSCs. Given this result, it is perhaps even more surprising that adipogenesis of H1 MSC was significantly promoted in 3D composites and was further enhanced in the composite containing ColI.

### ECM composites attenuate *IL-6* gene expression

**FIG. 5.**
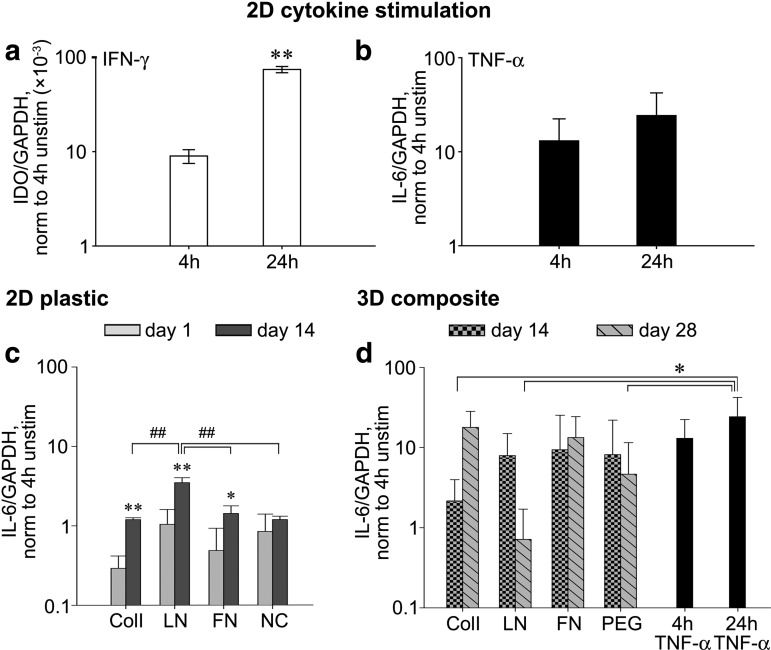
The expression of *IL-6* and *IDO* genes by hMSCs was significantly upregulated both in 2D cytokine stimulation and in 3D ECM composites. Both indoleamine 2,3-dioxygenase (*IDO*) and interleukin 6 (*IL-6*) were significantly upregulated 24 h after IFN-γ **(a)** and TNF-α **(b)** stimulation in 2D cultures, respectively. *IL-6* genes were distinctively upregulated in ECM-specific manner over 14 and 28 days in 2D and 3D culture, respectively **(c, d)**, while *IDO* genes were undetectable. Mean ± SD, *n* = 3. Student's *t*-test or ANOVA Fisher's LSD *post hoc* test, **p < 0.01 and *p < 0.05. ANOVA Tukey's HSD *post hoc* test, ##*p* < 0.01. * or ** without brackets between the time points in the group.

To determine whether immunomodulatory function of MSCs might also be altered with 3D exposure to ECM, we again capitalized on our composite system. hMSCs were entrapped in composites containing ColI, LN, or FN and cultured for 28 days and then probed for transcript expression of indoleamine 2,3-dioxygenase (*IDO*) and interleukin 6 (*IL-6*), two cytokines frequently associated with MSC immunomodulation.^25–28^ Both *IDO* and *IL-6* expression were inducible by IFN-γ and TNF-α stimulation in 2D culture, respectively (Fig. 5a, b). In 3D, expression of *IDO* gene was undetectable, similar to unstimulated MSC expression in 2D (data not shown). The expression of *IL-6* on 2D ECM films was significantly increased relative to unstimulated hMSC for 14 days except NC control (Fig. 5c). However, the expression of *IL-6* in 3D ECM composites was significantly increased relative to unstimulated hMSC expression in 2D. And in fact, expression was modulated in an ECM-specific manner. At 28 days, composites containing ColI or FN maintained levels of *IL-6* gene expression statistically similar to that of 2D hMSC cultures with TNF-α stimulation (24 h, Fig. 5d). At the same time point, composites without ECM and those containing LN exhibited *IL-6* expression above that of 2D unstimulated MSCs, but less than levels with TNF-α stimulation. Collectively, the expression of *IL-6* was elevated in 3D spaces for several days and the elevation of *IL-6* production in 3D was modulated to varying degrees by the type of ECM supplied exogenously at day 0.

## Discussion

Here, we show for the first time the impact of individual, intact ECM proteins presented in 3D on MSC functional potential both in terms of differentiation and immunomodulation. This work is important for those attempting to develop 3D ECM-containing delivery vehicles for MSC transplantation and for predicting behavior of transplanted cells to ECM-rich microenvironments. Previously, our laboratory showed that 2D ECM coatings modulated the differentiation of bone marrow-derived MSCs into different lineages in an ECM-specific manner over 14 days, comparable to chemical induction.^1^ But extension of these studies to 3D has been challenging because, with the exception of type I collagen, hyaluronan, and fibrin, ECM proteins isolated from tissues cannot reassemble reliably into robust 3D scaffolds capable of supporting cellular populations. Type I collagen has been utilized as a supporting scaffold for other ECM proteins purified from tissue^6,29^ or lyophilized ECM mixtures extracted from decellularized tissue.^30^ However, in these cases it is challenging to distinguish biochemical effects of the added ECM components from that of the supporting ECM (i.e., type I collagen) scaffold. Recently, our laboratory designed a synthetic-natural biomaterial *composite* that can present whole-molecule ECM proteins to cells without chemically cross-linking ECM proteins to the 3D biomaterial platform, preserving native secondary and tertiary structures necessary for appropriate biochemical signaling.^18^ In addition, since the impact of substrate stiffness on MSC differentiation is now well appreciated,^31,32^ the composite platform was designed to maintain consistent macroscale storage modulus (0.9 kPa) even with alterations in ECM type.^18^ Finally, the 3D scaffold was verified to maintain diffusion of well-characterized bovine serum albumin molecules in the same order of magnitude (Supplementary Table S1). These controls allowed us to compare functional outcomes of MSC exposure to individual ECM in 3D and relate those outcomes to findings in 2D. Like 2D, ECM proteins alone could stimulate multi-lineage differentiation in 3D at levels approaching and sometimes exceeding those attained with standard chemical differentiation protocols. Unlike 2D, adipogenic differentiation was favored with ECM serving to refine this response.

Adipogenic differentiation of MSCs and other multipotent cells has been a great interest to endocrinologists, stem cell biologists, and tissue engineers. The adipose tissue is an endocrine/paracrine regulator of energy metabolism and dysregulation can lead to type II diabetes and cardiovascular disorders among others. From a tissue engineering standpoint, burn patients suffering from soft tissue atrophy or facial lipodystrophy from antiviral medications for human immunodeficiency virus would benefit soft tissue reconstruction. Despite the importance of adipose tissue model systems and regeneration, mechanistic understanding of adipogenesis is still developing and methods to derive adipocytes from stem cells are quite variable.^33^ Further, the role of ECM proteins and three dimensionality has only recently been appreciated.^9^ Indeed, human mesenchymal progenitor cell spheroids formed in 2–3 μm honeycomb scaffolds significantly upregulate *PPARG*, *CEBPA* (CCAAT-enhancer-binding protein α, C/EBP-α), *ADIPOQ* (adiponectin), and *ALBP* (fatty acid-binding protein 4, FABP4, or aP2) gene expressions over 2D culture scenarios after only 3 days.^34^ This example highlights the importance of three dimensionality for adipogenesis, but exploits chemical induction without exogenous ECM proteins.

When ECM proteins are added the induction protocol for adipogenesis, augmented differentiation is observed. For example, human MSC spheroids cultured in rotating wall vessel bioreactor in combination with adipogenic induction showed significantly enhanced *PPARG*, *CEBPA*, *ALBP*, and *LPL* (lipoprotein lipase) 7 days after culture initiation compared with 2D culture.^35^ Further, covalent conjugation of FN on 2D polyacrylamide soft (∼0.6 kPa) substrate also significantly improved the expression of *CEBPA* and *LPL* over 10 days with microscale circular patterns (∼1000 μm^2^) relative to 2D polyacrylamide substrate without patterns.^7^ This is an interesting result as our composites are on the order of 0.9 kPa in stiffness, suggesting the possibility that 3D mechanical properties in addition to ECM exposure further enable adipogenesis. The impact of mechanical properties on MSC differentiation is well characterized in 2D^31^ and 3D,^36^ where adipogenic or neurogenic commitment occurs in softer environments at levels dependent on substrate/scaffold materials. Also of note, intramuscular preadipocytes (BIP) have been reported to produce collagens type I, V, VI, and fibronectin at high levels *in vitro*, while type IV collagen and laminin were produced at lower levels.^37^ The ECM-niche of fat then may promote adipogenesis and explain why composites with LN saw lower levels of differentiation.

Composites without ECM proteins were unable to maintain adipogenic differentiation after 14 days, supporting a necessity of not only the third dimensionality and associated mechanical properties but also ECM proteins for adipogenesis. Like other connective tissue cell types with mesenchymal origin, adipocytes are surrounded by collagens and basal lamina containing ECM proteins.^38^ Since only a lipid monolayer forms the boundary between stored fat and cytosol, transfer of mechanical stress from the outside to the inside of the adipocyte can be decreased by adipocyte ECM proteins and also redistribute forces over a larger area of tissue.^39^ These findings reflect the critical requirement of adipocyte ECM proteins for the development of preadipocyte to fat-storing adipocytes, in part explaining the attenuation of *PPARG* expression from composites with no ECM. To guide interpretation of these results, it should be noted that composites without exogenous ECM were less able to sustain cell viability long term. We previously reported a reduction of viability to as low as 60% over the course of several weeks in PEG (no ECM control) and thus differences in differentiation relative to the no ECM controls might relate to factors associated with cell death instead of or in addition to lack of signaling associated with exogenous ECM. For this reason we emphasized comparison to chemical induction and between ECM-containing composites.

In addition to multipotency of hMSCs, the immunomodulatory properties of MSCs have been exploited to treat multiple diseases including myocardial infarction^40,41^ or graft versus host disease^42^ to name a few. Although the exact mechanistic details are unclear, MSCs have been reported to be hypoimmunogenic, enabling MSC transplantation across major histocompatibility barriers and creating off-the-shelf therapies with allogenic MSCs. It should be noted that hESC-derived MSCs (both H1 and H9 MSCs) exhibit very similar immunosuppressive properties and multipotency relative to bone marrow-derived MSCs including the three main characteristics of MSCs including adherence to plastic, positive expression of CD29, CD44, CD54, CD73, CD90, and CD105, and negative expression of CD34, CD45, and CD31 and their ability to differentiate into osteogenic, adopogenic, and chondrogenic lineages.^20,43,44^ Here, we selected a small subset of cytokines to determine whether MSCs, by the engagement of ECM proteins in 3D, will modulate the expression of two immunomodulatory genes, *IDO* and *IL-6*. IDO (indoleamine-2,3-dioxygenase) is an enzyme that catalyzes the degradation of tryptophan along the metabolic pathway to kynurenine. MSC-mediated immunosuppression is believed to result from the depletion of tryptophan and the local accumulation of tryptophan metabolites,^45^ suppressing T cell and natural killer cell proliferation.^46,47^ As observed in Figure 5 and according to results using single cell RNA-sequencing of murine MSCs from our laboratory,^48^ MSCs normally express minimal levels of *IDO* while the *IDO* mRNA levels are found to be significantly elevated upon stimulation with inflammatory cytokines, primarily IFN-γ. IL-6 is known as a pleiotropic cytokine suppressing T-cell proliferation and local inflammation.^26,49^ While MSC-secreted IL-6 is reported to be proinflammatory,^50^ IL-6 can also be anti-inflammatory to modulate other proinflammatory cytokines.^51,52^ Although the inflammatory nature of IL-6 in 3D ECM composites was hard to define, the upregulation of *IL-6* gene in 3D is significant and is surprisingly sustained over multiple weeks (Fig. 5). Aggregates of MSCs showed a similar result after 7 days in culture^53^ and MSCs encapsulated in 3D hydrogels also modulated IL-6 secretion at day 14. In this case an association between IL-6 and hydrogel stiffness was observed within the softest hydrogel (1.5 kPa) showing the most significant upregulation of expression of *IL-6.*^10^ Future studies would be wise to assess how immunodulation of MSCs changes in the face of distinct ECM of different tissue types following transplantation.

## Conclusion

These results further elucidate the importance of ECM in guiding differentiation of stem cells in tissue and more surprisingly, associated immunomodulatory cytoine production. These data set the stage for synthetic biomaterial development for a multitude of applications including tuning of differentiation and immunomodulation to enable proactive delivery vehicles for stem cell transplantation.

## Supplementary Material

Supplemental data

## Abbreviations Used

BSA: bovine serum albumin
cDNA: complementary DNA
DABCO: 1,4-diazabicyclo[2,2,2]octane
DAPI: 4′,6-diamidino-2-phenylindole
ECM: extracellular matrix
FN: fibronectin
hESCs: human embryonic stem cells
hMSC: human mesenchymal stem cells
IDO: indoleamine 2,3-dioxygenase
IL-6: interleukin 6
LN: laminin
MSCs: mesenchymal stem cells
MPLSM: multi-photon laser scanning microscopy
NC: no construct
PBS: phosphate buffered saline
qRT-PCR: quantitative real-time polymerase chain reaction
RT: room temperature
TNF-α: tumor necrosis factor-α

## References

[1] Santiago JA, Pogemiller R, Ogle BM Heterogeneous differentiation of human mesenchymal stem cells in response to extended culture in extracellular matrices. Tissue Eng Part A. 2009;15:3911–3922 19911955 10.1089/ten.tea.2008.0603 PMC2792070

[2] Rao Pattabhi S, Martinez JS, Keller TCS III Decellularized ECM effects on human mesenchymal stem cell stemness and differentiation. Differentiation. 2014;88:131–143 25578478 10.1016/j.diff.2014.12.005 PMC4336570

[3] Prewitz MC, Seib FP, von Bonin M, et al. Tightly anchored tissue-mimetic matrices as instructive stem cell microenvironments. Nat Methods. 2013;10:788–794 23793238 10.1038/nmeth.2523

[4] Singh P, Schwarzbauer JE Fibronectin and stem cell differentiation–lessons from chondrogenesis. J Cell Sci. 2012;125:3703–3712 22976308 10.1242/jcs.095786 PMC3462078

[5] Hynes RO The Extracellular matrix: not just pretty fibrils. Science. 2009;326:1216–1219 19965464 10.1126/science.1176009 PMC3536535

[6] Yang F, Cho S-W, Son SM, et al. Combinatorial extracellular matrices for human embryonic stem cell differentiation in 3D. Biomacromolecules. 2010;11:1909–1914 20614932 10.1021/bm100357t PMC2946176

[7] Lee J, Abdeen AA, Zhang D, et al. Directing stem cell fate on hydrogel substrates by controlling cell geometry, matrix mechanics and adhesion ligand composition. Biomaterials. 2013;34:8140–8148 23932245 10.1016/j.biomaterials.2013.07.074

[8] Sa S, Wong L, McCloskey KE Combinatorial fibronectin and laminin signaling promote highly efficient cardiac differentiation of human embryonic stem cells. Biores Open Access. 2014;3:150–161 25126479 10.1089/biores.2014.0018 PMC4120929

[9] Baker BM, Chen CS Deconstructing the third dimension: how 3D culture microenvironments alter cellular cues. J Cell Sci. 2012;125:3015–3024 22797912 10.1242/jcs.079509 PMC3434846

[10] Marklein RA, Soranno DE, Burdick JA Magnitude and presentation of mechanical signals influence adult stem cell behavior in 3-dimensional macroporous hydrogels. Soft Matter. 2012;8:8113–8120

[11] Köllmer M, Keskar V, Hauk TG, et al. Stem cell-derived extracellular matrix enables survival and multilineage differentiation within superporous hydrogels. Biomacromolecules. 2012;13:963–973 22404228 10.1021/bm300332w PMC3322260

[12] Baraniak P, McDevitt T Scaffold-free culture of mesenchymal stem cell spheroids in suspension preserves multilineage potential. Cell Tissue Res. 2012;347:701–711 21833761 10.1007/s00441-011-1215-5 PMC4149251

[13] Higuchi A, Ling Q-D, Hsu S-T, et al. Biomimetic cell culture proteins as extracellular matrices for stem cell differentiation. Chem Rev. 2012;112:4507–4540 22621236 10.1021/cr3000169

[14] Tian XF, Heng BC, Ge Z, et al. Comparison of osteogenesis of human embryonic stem cells within 2D and 3D culture systems. Scand J Clin Lab Invest. 2008;68:58–67 18224557 10.1080/00365510701466416

[15] Riccio M, Resca E, Maraldi T, et al. Human dental pulp stem cells produce mineralized matrix in 2D and 3D cultures. Eur J Histochem. 2010;54:e46 21263745 10.4081/ejh.2010.e46 PMC3167326

[16] Ribeiro AS, Powell EM, Leach JB Neural stem cell differentiation in 2D and 3D microenvironments. presented at the 26th Southern Biomedical Engineering Conference, College Park, Maryland, USA, 422–425, 2010

[17] Martino MM, Mochizuki M, Rothenfluh DA, et al. Controlling integrin specificity and stem cell differentiation in 2D and 3D environments through regulation of fibronectin domain stability. Biomaterials. 2009;30:1089–1097 19027948 10.1016/j.biomaterials.2008.10.047 PMC2718049

[18] Jung JP, Sprangers AJ, Byce JR, et al. ECM-incorporated hydrogels cross-linked via native chemical ligation to engineer stem cell microenvironments. Biomacromolecules. 2013;14:3102–3111 23875943 10.1021/bm400728e PMC3880157

[19] Hu B-H, Su J, Messersmith PB Hydrogels cross-linked by native chemical ligation. Biomacromolecules. 2009;10:2194–2200 19601644 10.1021/bm900366e PMC2790863

[20] Trivedi P, Hematti P Derivation and immunological characterization of mesenchymal stromal cells from human embryonic stem cells. Exp Hematol. 2008;36:350–359 18179856 10.1016/j.exphem.2007.10.007 PMC2315792

[21] Trivedi P, Hematti P Simultaneous generation of CD34+ primitive hematopoietic cells and CD73+ mesenchymal stem cells from human embryonic stem cells cocultured with murine OP9 stromal cells. Exp Hematol. 2007;35:146–154 17198883 10.1016/j.exphem.2006.09.003

[22] Xu W, Zhang X, Qian H, et al. Mesenchymal stem cells from adult human bone marrow differentiate into a cardiomyocyte phenotype in vitro. Exp Biol Med (Maywood). 2004;229:623–631 15229356 10.1177/153537020422900706

[23] Chung C, Burdick JA Influence of three-dimensional hyaluronic acid microenvironments on mesenchymal stem cell chondrogenesis. Tissue Eng Part A. 2008;15:243–254 19193129 10.1089/ten.tea.2008.0067 PMC2678568

[24] Pankov R, Yamada KM Fibronectin at a glance. J Cell Sci. 2002;115:3861–3863 12244123 10.1242/jcs.00059

[25] Ma S, Xie N, Li W, et al. Immunobiology of mesenchymal stem cells. Cell Death Differ. 2014;21:216–225 24185619 10.1038/cdd.2013.158 PMC3890955

[26] Djouad F, Charbonnier L-M, Bouffi C, et al. Mesenchymal stem cells inhibit the differentiation of dendritic cells through an interleukin-6-dependent mechanism. Stem Cells. 2007;25:2025–2032 17510220 10.1634/stemcells.2006-0548

[27] Bernardo ME, Fibbe WE Mesenchymal stromal cells: sensors and switchers of inflammation. Cell Stem Cell. 2013;13:392–402 24094322 10.1016/j.stem.2013.09.006

[28] Wang Y, Chen X, Cao W, et al. Plasticity of mesenchymal stem cells in immunomodulation: pathological and therapeutic implications. Nat Immunol. 2014;15:1009–1016 25329189 10.1038/ni.3002

[29] Battista S, Guarnieri D, Borselli C, et al. The effect of matrix composition of 3D constructs on embryonic stem cell differentiation. Biomaterials. 2005;26:6194–6207 15921736 10.1016/j.biomaterials.2005.04.003

[30] Duan Y, Liu Z, O'Neill J, et al. Hybrid gel composed of native heart matrix and collagen induces cardiac differentiation of human embryonic stem cells without supplemental growth factors. J Cardiovasc Transl Res. 2011;4:605–615 21744185 10.1007/s12265-011-9304-0 PMC3196310

[31] Engler AJ, Sen S, Sweeney HL, et al. Matrix elasticity directs stem cell lineage specification. Cell. 2006;126:677–689 16923388 10.1016/j.cell.2006.06.044

[32] MacQueen L, Sun Y, Simmons CA Mesenchymal stem cell mechanobiology and emerging experimental platforms. J R Soc Interface. 2013;10:20130179 23635493 10.1098/rsif.2013.0179 PMC3673151

[33] Scott MA, Nguyen VT, Levi B, et al. Current methods of adipogenic differentiation of mesenchymal stem cells. Stem Cells Dev. 2011;20:1793–1804 21526925 10.1089/scd.2011.0040 PMC3182038

[34] Miyagawa Y, Okita H, Hiroyama M, et al. A microfabricated scaffold induces the spheroid formation of human bone marrow-derived mesenchymal progenitor cells and promotes efficient adipogenic differentiation. Tissue Eng Part A. 2010;17:513–521 20818998 10.1089/ten.TEA.2009.0810

[35] Frith JE, Thomson B, Genever PG Dynamic three-dimensional culture methods enhance mesenchymal stem cell properties and increase therapeutic potential. Tissue Eng Part C Methods. 2010;16:735–749 19811095 10.1089/ten.TEC.2009.0432

[36] Huebsch N, Arany PR, Mao AS, et al. Harnessing traction-mediated manipulation of the cell/matrix interface to control stem-cell fate. Nat Mater. 2010;9:518–526 20418863 10.1038/nmat2732 PMC2919753

[37] Nakajima I, Aso H, Yamaguchi T, et al. Adipose tissue extracellular matrix: newly organized by adipocytes during differentiation. Differentiation. 1998;63:193–200 9745710 10.1111/j.1432-0436.1998.00193.x

[38] Pierleoni C, Verdenelli F, Castellucci M, et al. Fibronectins and basal lamina molecules expression in human subcutaneous white adipose tissue. Eur J Histochem. 1998;42:183–188 9857243

[39] Mariman EM, Wang P Adipocyte extracellular matrix composition, dynamics and role in obesity. Cell Mol Life Sci. 2010;67:1277–1292 20107860 10.1007/s00018-010-0263-4 PMC2839497

[40] Huang X-P, Sun Z, Miyagi Y, et al. Differentiation of allogeneic mesenchymal stem cells induces immunogenicity and limits their long-term benefits for myocardial repair. Circulation. 2010;122:2419–2429 21098445 10.1161/CIRCULATIONAHA.110.955971

[41] Dhingra S, Li P, Huang X-P, et al. Preserving prostaglandin E2 level prevents pejection of implanted allogeneic mesenchymal stem cells and restores postinfarction ventricular function. Circulation. 2013;128:S69–S78 24030423 10.1161/CIRCULATIONAHA.112.000324

[42] Le Blanc K, Rasmusson I, Sundberg B, et al. Treatment of severe acute graft-versus-host disease with third party haploidentical mesenchymal stem cells. Lancet. 2004;363:1439–1441 15121408 10.1016/S0140-6736(04)16104-7

[43] Yen BL, Chang CJ, Liu K-J, et al. Brief report—Human embryonic stem cell-derived mesenchymal progenitors possess strong immunosuppressive effects toward natural killer cells as well as T lymphocytes. Stem Cells. 2009;27:451–456 18988708 10.1634/stemcells.2008-0390

[44] Sánchez L, Gutierrez-Aranda I, Ligero G, et al. Enrichment of human ESC-derived multipotent mesenchymal stem cells with immunosuppressive and anti-inflammatory properties capable to protect against experimental inflammatory bowel disease. Stem Cells. 2011;29:251–262 21732483 10.1002/stem.569

[45] Mellor AL, Munn DH IDO expression by dendritic cells: tolerance and tryptophan catabolism. Nat Rev Immunol. 2004;4:762–774 15459668 10.1038/nri1457

[46] Meisel R, Zibert A, Laryea M, et al. Human bone marrow stromal cells inhibit allogeneic T-cell responses by indoleamine 2,3-dioxygenase–mediated tryptophan degradation. Blood. 2004;103:4619–4621 15001472 10.1182/blood-2003-11-3909

[47] Spaggiari GM, Capobianco A, Abdelrazik H, et al. Mesenchymal stem cells inhibit natural killer-cell proliferation, cytotoxicity, and cytokine production: role of indoleamine 2,3-dioxygenase and prostaglandin E2. Blood. 2008;111:1327–1333 17951526 10.1182/blood-2007-02-074997

[48] Freeman BT, Jung JP, Ogle BM Single-cell RNA-seq of bone marrow-derived mesenchymal stem cells reveals unique profiles of lineage priming. PLoS One. 2015;10:e0136199 26352588 10.1371/journal.pone.0136199 PMC4564185

[49] Bouffi C, Bony C, Courties G, et al. IL-6-dependent PGE2 secretion by mesenchymal stem cells inhibits local inflammation in experimental arthritis. PLoS One. 2010;5:e14247 21151872 10.1371/journal.pone.0014247 PMC2998425

[50] Aggarwal S, Pittenger MF Human mesenchymal stem cells modulate allogeneic immune cell responses. Blood. 2005;105:1815–1822 15494428 10.1182/blood-2004-04-1559

[51] Xing Z, Gauldie J, Cox G, et al. IL-6 is an antiinflammatory cytokine required for controlling local or systemic acute inflammatory responses. J Clin Invest. 1998;101:311–320 9435302 10.1172/JCI1368 PMC508569

[52] Scheller J, Chalaris A, Schmidt-Arras D, et al. The pro- and anti-inflammatory properties of the cytokine interleukin-6. Biochim Biophys Acta. 2011;1813:878–888 21296109 10.1016/j.bbamcr.2011.01.034

[53] Tsai A-C, Liu Y, Yuan X, et al. Compaction, fusion, and functional activation of three-dimensional human mesenchymal stem cell aggregate. Tissue Eng Part A. 2015;21:1705–1719 25661745 10.1089/ten.tea.2014.0314 PMC4426301

