# Supplementary material for: Heterogeneous Differentiation of Human Mesenchymal Stem Cells in 3D Extracellular Matrix Composites
Source: Biores Open Access. 2016 Jan 1;5(1):37–48. doi: 10.1089/biores.2015.0044 (PMC4744874; doi:10.1089/biores.2015.0044)
Supplement: Supplemental data [file Supp_Data.pdf]

Supplementary Data

Methods and Materials

Culture of human mesenchymal stem cells

α-MEM (cat# 12000-022; Life Technologies) medium supplemented with 1% nonessential amino acids (cat# 11140-050; Life Technologies) and 10% fetal bovine serum (FBS, cat# 30070.03; Thermo Scientific) at 37°C/5% CO_2. Human mesenchymal stem cells (hMSCs) were plated at 4.0×10^5 cells/175 cm^2 and harvested at 60–70% confluence. α-MEM medium was changed one day after plating and every 2 days thereafter. hMSCs were harvested (passages from 9 to 10) via application of 0.25% Trypsin in HBSS (cat# 25-054-CI; Mediatech) and pelleted using a centrifuge at 300 *g* for 5 min. Supernatant was removed and hMSCs were resuspended in fresh medium and were plated to gelatin (0.1%)-coated plates.

Flow cytometry

H1 MSCs were trypsinized at passage 8, centrifuged at 300 *g* for 5 min. These cells were resuspended in phosphate-buffered saline (PBS) and centrifuged at 300 *g* for 5 min to rinse. The cell pellet was resuspended in 2 mL of 4% paraformaldehyde (PFA) in PBS for 15 min at room temperature (RT). These cells were centrifuged at 300 *g* for 5 min and resuspended in 500 μL PBS. About 10 μL primary antibodies were added to the cell suspension in 500 μL PBS, followed by 1 h incubation at RT. Cells were centrifuged and resuspended in 500 μL PBS to rinse, followed by resuspension of 100 μL PBS. Secondary antibodies (1:200 dilution) were added to the 100 μL cell suspension, followed by 30 min incubation at RT. Cells were washed with 500 μL PBS and resuspended in 500 μL PBS containing 10% FBS for flow cytometry. Cell surface staining was analyzed using SLR II H1160 (Becton Dickinson). FlowJo software version 7.6 (FlowJo) was used to analyze the acquired data. Antibodies used were goat anti-CD73 (cat# sc-14682; Santa Cruz Biotechnology), rabbit anti-CD90 (cat# AP2050a; Abgent), goat anti-CD105 (cat# AF1097; R&D Systems), donkey anti-goat FITC (cat# A11055; Life Technologies), and goat anti-rabbit FITC (cat# 31583; Thermo Scientific).

Supplementary Table S1. Effective Diffusion Coefficients (D_e) of ECM Composites at 37°C

ECM composites	$\times10^{-7}$ (cm^2/s)
Collagen type I	1.88 ± 0.20
Laminin	1.73 ± 0.08
Fibronectin	2.96 ± 0.07
PEG (no ECM)	3.12 ± 0.77

Diffusion of BSA in water at 37°C is 9.14×10^{-7} cm^2/s.[7]
BSA, bovine serum albumin; ECM, extracellular matrix.

Formation of ECM composites

Cysteine- and thioester-terminated four-armed PEG macromonomers were synthesized using previously published protocols.[1] To produce an ECM composite, PEG-Cys was dissolved in α-MEM, while PEG-thioester was dissolved in α-MEM supplemented with additional $NaHCO_3$ to adjust the final concentration of $NaHCO_3$ to 35 mM. Either rat tail collagen type I (ColI, cat# 354236; Corning), mouse laminin (LN, cat# 354259; Corning), or human plasma fibronectin (FN, cat# 356008; Corning) was neutralized and polymerized at 37°C/5% CO_2. In each neutralization and mixing step, the pH of the mixtures was verified by pH indicator strips (from pH 5.0 to 10.0 with 0.5 increment, cat# 9588; EMD Millipore), confirming that the mixture pH was between 7.5 and 8.0. For encapsulation, 1×10^5 hMSCs were transferred to an Eppendorf tube, cells were centrifuged at 200 *g* for 5 min, resuspended with either viscous ColI, LN, or FN solutions and incubated for 15 min at 37°C/5% CO_2. This cell–ECM mixture was then combined with both PEG precursors, followed by casting these viscous solutions onto sterilized dialysis tubes (cat# 69562; Thermo Scientific) with a 200 μL blunt pipette tip. ECM composites were cross-linked without immersing dialysis tubes on a 48-well plate for 30 min at 37°C/5% CO_2, then moved to a well containing 500 μL of fresh cell culture medium on a 48-well plate. This procedure was repeated three times at 30 min intervals, followed by transferring ECM composites to a well on a 24-well plate containing 1 mL of fresh medium. The final concentrations of PEG and ECM were 40 and 2 mg/mL, respectively, for all subsequent studies. ECM composites were kept in α-MEM medium at 37°C/5% CO_2 for up to 28 days (medium change every 2 day).

Imaging ECM composites using multi-photon laser scanning microscopy

At days 1, 14, and 28, ECM composites were fixed with 4% PFA for 15 min and then permeabilized with 0.1% Triton-X in 1% bovine serum albumin (BSA) in PBS for 15 min on a rocker at RT. Following incubation overnight at 4°C in blocking buffer (5% BSA with 10% goat serum in PBS), ColI, LN, and FN were probed with rabbit anti-collagen type I (cat# AB755P; EMD Millipore), rat anti-laminin-1 A & B chains (cat# MAB1904; Chemicon), and rabbit anti-fibronectin

(cat# ab23750; Abcam) antibodies, respectively. The primary antibodies were diluted at a 1:80 dilution in blocking buffer and incubated for 1 h on a rocker at RT. The antibodies against ColI, LN, or FN were detected by incubating with a secondary antibody, goat anti-rabbit FITC-conjugated (cat# 31583; Thermo Fisher Scientific), or goat anti-rat FITC-conjugated (cat# 3010-02; Southern Biotech) at a 1:100 dilution in blocking buffer for 45 min on a rocker at RT, followed by incubation with TRITC-conjugated phalloidin (cat# FAK100; EMD Millipore) at a 1:100 dilution in blocking buffer for 45 min on a rocker at RT. Nuclei in ECM composites were stained with 100 ng/mL 4′,6-diamidino-2-phenylindole (DAPI, cat# D9542; Sigma-Aldrich) in water. ECM proteins, nuclei, and actin filaments were visualized with a 40× (NA = 0.8) objective lens by using a mode-locked Ti:Sapphire laser on a multi-photon laser scanning microscope (Bruker Nano). 3D reconstructions were generated using Imaris 7.5.2 software (Bitplane, Inc.).

Extracting RNA from ECM composites and hMSC cultures

At days 0, 1, 14, or 28, ECM composites were collected in an Eppendorf tube and stored at −80°C with 10 μL of TRIzol® (cat# 15596-026; Life Technologies) per ECM composite until further mechanical disruption. When thawed, additional 30 μL of TRIzol was added to each ECM composite and mechanically disrupted by micropestles. After ground ECM composites were allowed to stand for 5 min at RT, 8 μL of chloroform was added and vigorously shaken by hand for ∼15 sec. After 3 min at RT, samples were centrifuged at 12,000 *g* for 15 min at 4°C, separating mixtures into three phases. Only a colorless upper aqueous phase was removed and transferred into a new Eppendorf tube. An equal volume of 70% ethanol (molecular biology grade) was added to each tube to make mixture of RNA in 35% ethanol. These mixtures were processed in PureLink RNA Mini Kit (cat# 12183-018A; Life Technologies) by following the protocol. From the stimulation experiments, hMSCs were removed from wells and resuspended in Lysis Buffer from the PerfectPure RNA Cultured Cell Kit (cat# 2302340; 5 Prime). Instructions from the kit were followed and RNA was eluted from the column in 50 μL of Elution Buffer.

hMSC chemical induction

For osteogenic differentiation, hMSCs were grown in α-MEM/10% FBS media containing osteogenic supplements (0.1 μM dexamethasone, 10 mM β-glycerolphosphate, and 200 μM ascorbic acid) with medium change every 3 days over 21 days.[2] For adipogenic differentiation, hMSCs were grown in α-MEM/10% FBS media containing adipogenic supplement (1 μM dexamethasone, 0.5 mM methyl-isobutylxanthine, and 10 U/mL insulin) with media changes every 3 days over 21 days.[2] For chondrogenic differentiation, hMSCs were grown in α-MEM/10% FBS media containing chondrogenic supplements (10 ng/mL transforming growth factor-β3[3] and 200 μM ascorbic acid) as a dense cell mass incubated at 37°C/5% CO_2 in 15 mL conical tubes with the caps slightly open.[2,3] Medium was changed every 3 days without disturbing the cell mass over 21 days. For cardiomyogenic differentiation, hMSCs were plated in α-MEM/10% FBS media for 24 h, which was changed to α-MEM/15% FBS media containing cardiomyogenic supplements (10 μM 5-azacytidine and 10 ng/mL basic fibroblast growth factor) for 24 h.[4] After this period, hMSCs were washed with PBS and the medium was changed to α-MEM/10% FBS media. Medium was changed every 3 days over 28 days. RNAs were extracted by adding Lysis Buffer from PureLink RNA Mini Kit (cat# 12183-018A; Life Technologies).

Adipogenic induction of H9 MSCs, immunofluorescence staining of FABP4 and PPAR-γ, and immunohistochemical Oil Red O staining

Mesenchymal stem cells were derived from H9 human embryonic stem cells.[5] H9 MSCs were cultured over 21 days with and without adipogenic induction. For adipogenic differentiation, hMSCs were grown in α-MEM/10% FBS media containing adipogenic supplement (1 μM dexamethasone, 0.5 mM methyl-isobutylxanthine, and 10 U/mL insulin) with media changes every 3 days.[2] After 21 days of culture initiation, H9 MSCs were fixed with 4% PFA for 15 min and permeabilized with 0.1% Triton-X 100/1% BSA for 15 min at RT. Each step was performed on a rocker, followed by 5 min washing with PBS. Following a 2 h incubation at RT on a rocker with blocking solution (10% goat serum/5% BSA), rabbit anti-FABP4 (cat# PA5-30591; Thermo Scientific, 1:100 dilution in the blocking solution) or rabbit-PPARγ (cat# PA3-821A; Thermo Scientific, 1:100 dilution in blocking solution) antibodies were incubated for 2 h, which were probed by goat anti-rabbit FITC (cat# 31583; Pierce, 1:200 dilution in blocking solution) for 45 min. Fixed H9 MSCs were stored in 2.5% anti-fade medium (1,4-diazabicyclo[2,2,2]octane (DABCO, cat# D27802; Sigma-Aldrich) in 1:1 PBS and glycerol) with 100 ng/mL DAPI

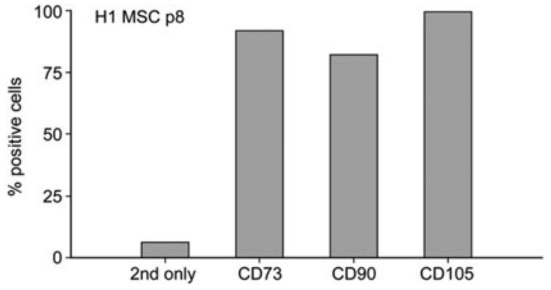

SUPPLEMENTARY FIG. S1. Flow cytometric analysis of human mesenchymal stem cells (hMSCs) derived from H1 ESCs. hMSCs were probed for expression of markers indicative of the MSC phenotypes, which were found to express 82% or higher levels of CD73, CD90, and CD105.

(cat# D9542; Sigma-Aldrich) until imaging. Fat droplets were stained by following the manufacturer's protocol (cat# KTORO; American MasterTech). H9 MSCs were imaged with an Olympus IX81ZDC microscope equipped with 10× (NA = 0.25) objective lens.

Measuring effective diffusion coefficients

ECM composites were prepared using the same methods and materials except the concentration of ECM proteins and the inclusion of BSA (cat# SH30574.01; Fisher Scientific). The final concentrations of ECM proteins and BSA were 1.9 and 1.0 mg/mL, respectively. ECM composites were kept in PBS at 37°C/5% CO_2 for 24 h. The concentrations of released BSA from ECM composites were measured using the BCA protein assay kit (cat# 23227; Pierce). The effective diffusion coefficients were calculated using the method described from Reference.[6]

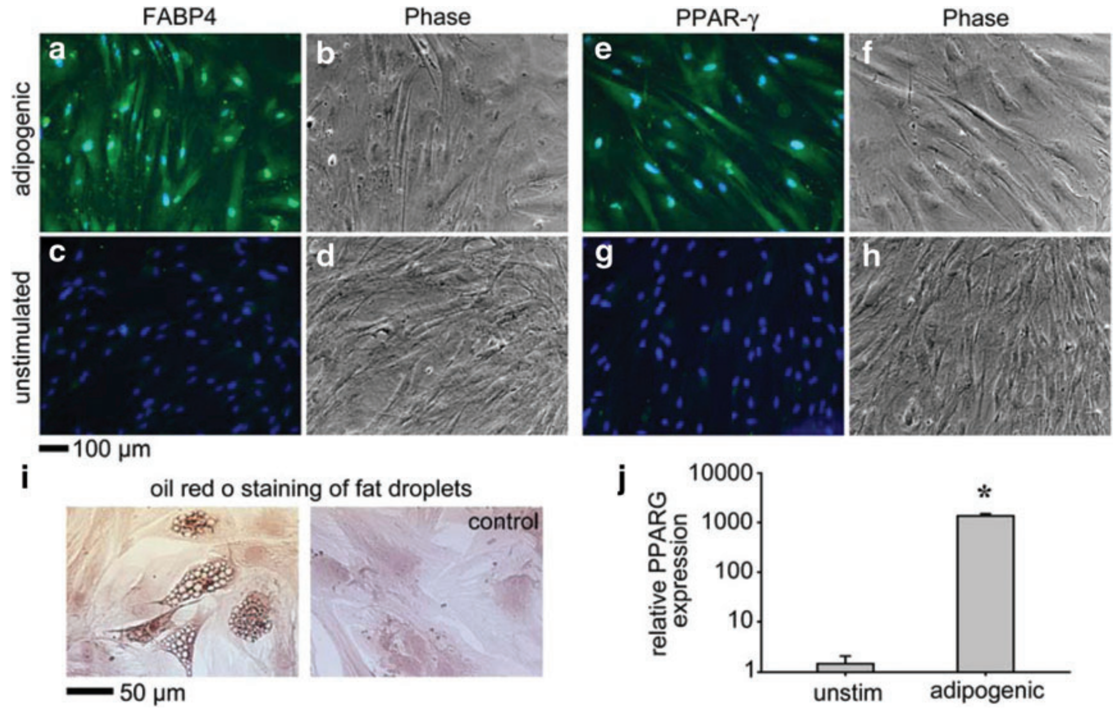

SUPPLEMENTARY FIG. S2. Immunofluorescence staining of FABP4 **(a–d)** and PPAR-γ **(e–h)** with adipogenic induction of H9 MSCs on 2D plastics for 21 days. These regions of interest stained positive for FABP4 **(a, b)** and PPAR-γ **(e, f)**. Green (FITC-conjugated secondary antibodies), blue (DAPI) **(c, d, g, h)**. Oil Red O staining of H9 MSCs with adipogenic induction for 21 days with control **(i)**. The expression of *PPARG* gene was measured by quantitative real-time polymerase chain reaction (qRT-PCR) after 21 days, normalized to the *PPARG* expression at day 0 **(j)**. Mean ± SD, $n = 3$, $*p < 0.01$ (Student's t-test, $\alpha = 0.05$).

Supplementary References

1. Jung JP, Sprangers AJ, Byce JR, et al. ECM-incorporated hydrogels cross-linked via native chemical ligation to engineer stem cell microenvironments. Biomacromolecules. 2013;14:3102–3111.
2. Trivedi P, Hematti P. Simultaneous generation of CD34+ primitive hematopoietic cells and CD73+ mesenchymal stem cells from human embryonic stem cells cocultured with murine OP9 stromal cells. Exp Hematol. 2007;35:146–154.
3. Chung C, Burdick JA. Influence of three-dimensional hyaluronic acid microenvironments on mesenchymal stem cell chondrogenesis. Tissue Eng Part A. 2008;15:243–254.
4. Xu W, Zhang X, Qian H, et al. Mesenchymal stem cells from adult human bone marrow differentiate into a cardiomyocyte phenotype in vitro. Exp Biol Med. 2004;229:623–631.
5. Trivedi P, Hematti P. Derivation and immunological characterization of mesenchymal stromal cells from human embryonic stem cells. Exp Hematol. 2008;36:350–359.
6. Leach JB, Schmidt CE. Characterization of protein release from photo-crosslinkable hyaluronic acid-polyethylene glycol hydrogel tissue engineering scaffolds. Biomaterials. 2005;26:125–135.
7. Han JH, Krochta JM, Hsieh Y-L, et al. Mechanism and characteristics of protein release from lactitol-based cross-linked hydrogel. J Agric Food Chem. 2000;48:5658–5665.
